# Organizational strategies of eldercare work and health – Is the daily number of residents cared for over 14 months associated with back pain?

**DOI:** 10.5271/sjweh.4207

**Published:** 2025-05-01

**Authors:** Christian Tolstrup Wester, Stavros Kyriakidis, Anders Dreyer Frost, Charlotte Diana Nørregaard Rasmussen, Andreas Holtermann, David M Hallman

**Affiliations:** 1 The National Research Centre for the Working Environment, Lersø Parkallé 105, 2100 København Ø, Denmark.; 2 Department of Sports Science and Clinical Biomechanics, University of Southern Denmark, Odense, Denmark.; 3 Department of Occupational Health, Psychology and Sport Sciences, University of Gävle, Gävle, Sweden.

**Keywords:** eldercare worker, healthcare, follow-up study, staff ratio, workload distribution

## Abstract

**Objectives:**

The growing care demands of an aging population and a smaller workforce is a big societal problem. Therefore, knowledge on how to organize eldercare work without hampering workers' health is needed. We aimed to investigate if workers' daily number of residents cared for over 14 months is associated with low-back pain in eldercare workers.

**Methods:**

We included 513 eldercare workers from 122 wards. In each ward, we gathered quarterly data over 14 months on the number of residents, workers, and work schedules and calculated the daily numbers of residents each worker cared for. Workers reported intensity and days with low-back pain via monthly text messages over 14 months. Using generalized linear mixed models adjusted for confounders, we investigated the association between the number of residents workers cared for daily and low-back pain among those workers.

**Results:**

In 3-month periods over 14 months, caring for ≥1 resident per day was associated with a 4% [95% confidence interval (CI) 1.02–1.07] increased risk of more days with low-back pain, and a 2% (95% CI 1.00–1.03) increase in low-back pain intensity among workers.

**Conclusions:**

Eldercare workers are at a higher risk of experiencing low-back pain during periods when they care for a greater number of residents each day. Maintaining a consistent number of residents and workload for workers over a 14-month period could serve as an effective organizational strategy to prevent low-back pain.

The aging population (1) imposes considerable challenges for the eldercare sector in terms of retention and recruitment of staff to meet the increasing demands for care (2, 3). Consequently, eldercare workers might face a lower staffing ratio, where they need to provide care to a larger number of residents per work shift.

Giving care to more residents per work shift (lower staffing ratio) can lead to higher psychosocial and physical work demands on each worker, such as increased total quantitative demands and more resident handlings (4, 5). High psychosocial and physical work demands can in turn increase the risk of poor health among eldercare workers (6). On the contrary, a recent study did not find a ward-level staffing ratio to be associated with the health of eldercare workers (7). These contrary results may be explained by limited variation in staffing ratios between wards and insufficient analytical consideration of how residents were distributed among workers.

However, the current knowledge on the importance of staffing ratio for workers' health is based on cross-sectional study designs and self-reported information on staffing ratio (5, 7, 8). Moreover, eldercare homes are often organized in separate wards, differing in organizational characteristics, which likely influence how eldercare work (ie, resident care) is organized between the workers within the wards. As such, it is important to analytically embed that eldercare work is "nested" within wards and that workers within these wards care for a varying number of residents over time. Daily work schedules over 14 months can provide this data.

Among eldercare workers, low-back pain is a particularly prevalent musculoskeletal health issue (9). Low-back pain is the dominating cause of sick leave, early withdrawal from the labor market (10–12), and the main cause of years lived with disability (13). Thus, it is crucial to find solutions for the organization of eldercare work – in a situation of many residents and few workers – to improve prevention of low-back pain.

When reducing the number of residents per worker is not a feasible solution, two potential organizational strategies can help plan eldercare work to prevent 'overload' – a situation where the workload exceeds a worker's mental or physical capacity – and, in turn, reduce the risk of low-back pain. The first strategy focuses on evenly distributing the number of residents needing care among workers within wards. This approach is referred to as the 'even between-worker, within-ward strategy' (14). The second strategy emphasizes spreading the workload more evenly over a 14-month period for each worker to avoid periods of overload. This approach is known as the 'even between-period, within-worker strategy'.

However, very few studies have investigated how these organizational strategies are associated with workers' health by distinguishing between the effects of work demands at different levels of the organization. For example, the number of residents each worker cares for per day is likely to vary between wards, between workers within a ward (eg, some workers are assigned more residents than others) and between periods over 14 months for the individual worker (eg, a worker may be assigned more residents during a period to compensate for a shortage of staff). Consequently, the differences in exposure across the levels of the organization may respectively be associated with low-back pain. This knowledge can be valuable for designing feasible and effective organizational interventions, such as strategies targeting the distribution of workload among workers within a ward (15) or between periods over 14 months for the individual worker (16–18). Such studies can be crucial to understand how work can be organized to prevent pain in eldercare as well as in other occupational groups.

Therefore, we aimed to investigate if the daily number of residents cared for – between workers (within ward) and between-periods (within worker) over 14 months – are associated with low-back pain among eldercare workers. A 'period' consists of three months, and this study included five periods in total. Each period included daily work schedules and monthly measures of low-back pain, respectively.

## Methods

### Study design

**Figure 1:**
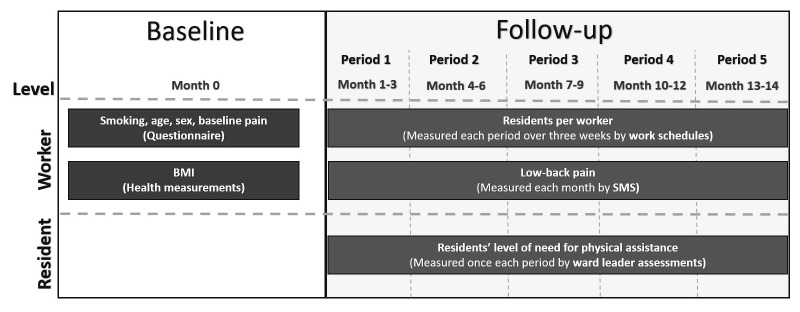
Data collection process, including repeated measurements at follow-up of exposure (ie, residents cared for per day among workers and level of need for physical assistance among the residents) and outcome (ie, low-back pain).

We used data from the Danish Observational Study of Eldercare Work and Musculoskeletal Disorders (DOSES). DOSES is an observational prospective workplace cohort designed to examine associations between physical and psychosocial working conditions, musculoskeletal pain and its consequences among eldercare employees at Danish nursing homes located in the Copenhagen area. Data were collected from September 2013 to January 2016. A detailed description of the cohort is provided elsewhere (19). This study uses baseline data and follow-up information over approximately 14 months for the exposure and outcome measures. We used a prospective design with repeated measures every three months, to assess the daily number of residents cared for over three weeks (exposure), and monthly to check the occurrence of low-back pain (outcome) among workers. This evaluation spanned 14 months, comprising four 3-month periods and one 2-month period (figure 1). The Danish Data Protection Agency and the regional Ethics Committee for the Capital Region of Denmark granted DOSES ethical approval (H-4-2013-028), and written informed consent was obtained from all eldercare workers before their involvement in the study.

### Study population

We invited 83 nursing homes located in Zeeland in the larger Copenhagen area in Denmark to participate in the study. These were purposively selected with the aim to include both smaller and larger nursing homes and different care models. Of these nursing homes, 20 agreed to participate (2 private and 18 municipal) and had an average of 6.3 wards [standard deviation (SD) 3.1], 79 residents (SD 28.9) and 70 eldercare workers (SD 27.7) who were employed >15 hours per week on day and evening shifts, with ≥25% of their time spent on direct resident care (19).

**Figure 2:**
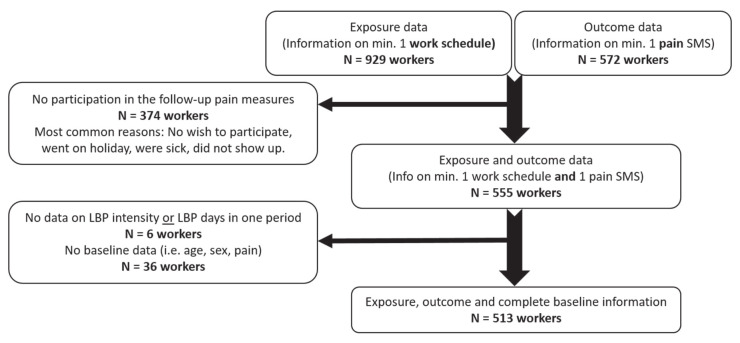
Flow diagram of the study participation.

A total of 941 eldercare workers were eligible for participation. Every third month, a responsible worker from each ward collected the work schedules of eligible workers, regardless of their acceptance of participation in the individual measurements. Information on minimum one work schedule was obtained for 929 workers and 1339 residents (figure 2). Of these, 572 workers responded to ≥1 follow-up SMS regarding low-back pain. Among the 379 workers (40%) who did not participate in the SMS-based low-back pain measurements, the most common reason was a lack of interest in participating, while other frequent reasons included being on holiday, being sick, or failing to show up. Ultimately, 555 workers (aged 19–71 years) provided data on both work schedules and low-back pain during follow-up.

Workers were excluded if they lacked information on follow-up measures on the number of days with or intensity of low-back pain (N=6) or any of the baseline measures (N=36) [ie, age, sex, body mass index (BMI), smoking, baseline low-back pain]. Therefore, a total of 513 workers across 122 eldercare wards and 20 nursing homes and 1161 residents were included in the final sample.

### Exposure variables

*Number of residents per day.* A worker in each ward was responsible for planning and reporting the individual work schedules every third month. The work schedules contained day-to-day information about which residents each worker cared for, and were collected for periods of 3 weeks (21 days), every third month over 14 months (see figure 1). The work schedule information was used to estimate the average number of residents each worker cared for per day, for each of the five periods.

Prior to the statistical analyses, we created three exposure variables following previously described procedures (20) to account for the variability in exposure (ie, the number of residents per day between wards, between workers (within-ward) and between periods (within-worker)). The detailed calculations of the exposure variables are described in the supplementary material, www.sjweh.fi/article/4207, description 1.

First, we calculated the ward mean (ie, the mean number of residents cared for per day for all workers in each ward), described in step 2 of the supplementary description 1. This variable represents the variation in exposure between wards, which was adjusted for in the analyses.

Second, we calculated the difference between the exposure for each worker and the ward mean (ie, worker-ward). This value was then averaged across all periods to obtain one value (worker-ward mean) for each worker at follow-up (step 4 in supplementary description 1). This variable represents the variation in exposure between workers (within-ward) and was used for further statistical analyses of 'the even between-worker, within-ward strategy' on the risk of low-back pain.

Third, to obtain a variable representing the change between periods (within-worker), we calculated the difference between the worker-ward mean and the worker-ward value for each period (step 5 in supplementary description 1). This value represents the variation in exposure between periods (within-worker) and was used for the analyses of 'the even between-period, within-worker strategy'.

### Outcome variable

*Low-back pain.* Eldercare workers were sent text messages (SMS) every four weeks over 14 months enquiring about the number of days they experienced low-back pain in the previous month ("In the last four weeks, how many days did you have pain in your low-back region?", response 0–28 days). If the workers replied that they had >0 days of pain, they were asked about the intensity of that pain ("On a scale from 0–10, what is the worst pain you have had in your lower back in the past four weeks?"), with answers ranging from 0 (no pain) to 10 (worst possible pain) (19). This scale was based on the standardized Nordic questionnaire for musculoskeletal symptoms (21). The workers answered the pain-related questions a maximum of 14 times and a minimum of 1 time.

### Potential confounders

Demographic and health-related data included age (years), sex (male or female), BMI (calculated from objectively measured height and weight obtained at the baseline health check), weekly work hours (time in hours) and smoking status [dichotomized to yes ("yes, daily", "yes, once in a while") and no ("no, but smoked in the past", "no, I have never smoked")]. We also included information on work ability based on a validated single item from the work ability index (22): "Imagine that your work ability is worth 10 points at its best. How many points would you give your current work ability?" [scale: 0 (unable to work) to 10 (best ability to work)]. Information about pain in the lower back was also obtained at baseline, as described in detail above regarding the low-back pain outcome ("In the last four weeks, how many days did you have pain in your low-back region?" (response range 0–28 days)).

### Statistical analyses

We used generalized linear mixed-effect models to investigate the association between the number of residents cared for per day (between workers and between-period, within-worker effects) and low-back pain (days and intensity). The purpose of the models was to estimate the risk of low-back pain by each unit change in the number of residents per day. To accurately model our data, we distinguished between variability in the number of residents per day at several levels: (i) between wards (covariate), (ii) between workers, within ward, (exposure 1) and (iii) between periods, within worker (exposure 2).

By adjusting for these levels of variability, we can more accurately estimate the risk of low-back pain related to number of residents cared for over time. Specifically, the 'between-worker effect' compares exposure between workers within the same ward, while the 'between-period, within-worker effect' reflects changes in each worker's exposure over time.

As such, for each of the two pain outcomes, we created two models: Model 1 included both exposures as fixed effects, as well as the ward mean to adjust for potential variability in exposure between wards. Model 2 also included age, sex, BMI, smoking, work ability, weekly work hours and baseline low-back pain (days or intensity depending on the specific model) as fixed effects. Moreover, to account for the clustering of data at both ward and worker levels in the outcome variables, random effects were included to capture variation between wards and between-workers. We used a negative binomial fit, due to the outcome variables (number of days and intensity of low-back pain) containing a high number of zeros and when fitted with Poisson distributions over-dispersion was detected (conditional mean < conditional variance). The negative binomial model was also supported by improved fit statistics, ie, the Akaike information criterion (AIC) values were lower for the final model. Moreover, missing data on the outcome variables at follow-up were considered missing at random as the missingness depended on observed but not unobserved variables. This allowed us to use all available data to estimate the fixed effects without imputations while maintaining valid inferences (23). Results are reported as incidence rate ratios (IRR) and 95% confidence intervals (CI), thus an alpha level of P<0.05 was considered statistically significant.

Moreover, we conducted two sensitivity analyses. In the first, we examined potential non-linear trends by grouping each of the two exposures into three categories: low (≤-1 resident daily), medium (>-1–<1 resident daily), and high (≥1 resident daily) (see supplementary table S2).

In the second sensitivity analyses, we created weighted scores for the number of residents cared for on a daily basis by accounting for the residents' level of need for physical assistance (see supplementary table S3). The resident's need for physical assistance (RNPA) scale consisted of four levels: light, moderate, extensive, and complete (24). Ward managers reported the RNPA for each resident in every ward and for each period. The RNPA scale has previously been validated against workplace observations, based on the same study population, showing good discriminative abilities for the number of resident handlings and duration (in minutes) of care situations (24). As such, the RNPA scale was used to calculate a weighted score for the number of residents per day, for each worker, in each ward, for each period, based on the mean duration of care situations for residents in each of the four RNPA levels (24) with light RNPA as the reference (see further description of the calculation in supplementary description 2). Then, based on the weighted score, we created the two exposure variables as previously described, reflecting the between-worker effect and the between-period, within-worker effect, and conducted analyses similar to the unweighted exposures. All analyses were conducted in RStudio v.4.2.2 (25) using the packages tidyverse (26), lme4 (27), blmeco (28), expss (29), flextable (30) and reshape (31).

## Results

### Descriptive results

We included 513 workers across 122 wards with 42 ward managers and 1161 nursing home residents. The workers responded on average to 10.2 of the 14 monthly text messages about low-back pain, while the wards provided data on work schedules on average 3.9 out of 5 periods and the RNPA scale on average 4.3 out of 5 periods, respectively. Table 1 shows the characteristics of the study sample at baseline, the number of residents per worker and low-back pain at baseline and follow-up. Workers cared on average for 4.3 residents per day (range 1–15). See supplementary table S1 for more descriptive information of the exposure variables (ie the between-worker effect and between-period, within-worker effect).

**Table 1:** Descriptive characteristics of eldercare workers across periods. [SD=standard deviation; RNPA=resident's need for physical assistance.]

		Overall (N=513 ^a^)	
		Mean (SD)	N (%)
Age		45.6 (10.9)	
Sex			
	Male		23 (4.4)
	Female		490 (95.5)
	Body mass index	26.7 (5.4)	
Smoking			
	Yes		179 (35.1)
	No		334 (65.1)
Work ability		8.6 (1.4)	
Weekly work hours		32.3 (3.7)	
Residents per day			
	Worker	4.3 (2.2)	
	Ward	4.4 (1.2)	
Residents per day (weighted) ^b^			
	Worker	7.1 (3.6)	
	Ward	7.4 (1.9)	
Residents per day with different needs for physical assistance ^b^			
	Light need	0.85 (0.9)	
	Moderate need	1.28 (1.2)	
	Extensive need	0.89 (0.9)	
	Complete need	1.13 (1.0)	
Low-back pain			
	Number of days (baseline)	8.0 (9.6)	
	Intensity (baseline)	4.0 (3.3)	
	Number of days (follow-up)	7.1 (8.5)	
	Intensity (follow-up) ^c^	3.5 (3.1)	

^a^ Unless anything else is specified, the sample includes 122 wards, 513 workers, and 5289 observations over 14 months. ^b^ The sample based on the RNPA scale consisted of 111 wards, 476 workers, and 4417 observations over 14 months. ^c^ N=1 missing in the main sample (N=512).

### Main analyses - Number of residents per day and low-back pain

Table 2 shows the associations between the number of residents per day, between workers (within-ward) and between periods (within-worker), and low-back pain over 14 months. For the between-period, within-worker effect, periods where workers cared for ≥1 resident per day were significantly associated with a 4% increased risk of reporting ≥1 day of low-back pain per month (adjusted model: IRR 1.04, 95% CI 1.02–1.07) and a 2% increase in the mean low-back pain intensity (adjusted model: IRR 1.02 95% CI 1.00–1.03), respectively. Results were similar in the unadjusted and adjusted models. For the between-worker effect, a higher number of residents per day compared to the ward mean was also positively associated with low-back pain. However, these associations were not statistically significant.

**Table 2:** Association between residents per day between-workers (within-ward) and between-periods (within-worker) and low-back pain (days and intensity) over 14 months at worker (N=513) and ward (N=122) levels. [IRR=incidence rate ratio; CI=confidence interval]

	Low-back pain (days)				Low-back pain (intensity)		
	Crude model ^a^		Adjusted model ^b^		Crude model ^a^		Adjusted model ^b^
	IRR (95% CI)		IRR (95% CI)		IRR (95% CI)		IRR (95% CI)
Residents per day							
Between-workers	1.04 (0.95–1.13)		1.06 (0.99–1.14)		1.00 (0.94–1.07)		1.03 (0.97–1.09)
Between-periods	1.05 (1.02–1.07) **		1.04 (1.02–1.07) **		1.02 (1.00–1.04) *		1.02 (1.00–1.03) *

Significance levels: *** < 0.001, ** < 0.01, * < 0.05, < 0.1 ^a^ Between-workers effect, between-periods within-worker effect, ward mean. ^b^ Crude model + age, sex, body mass index, smoking, work ability, weekly work hours, baseline low-back pain (number of days or intensity).

### Sensitivity analysis 1 – categorization of the exposures

In our first sensitivity analysis we examined a potential non-linear trend in the exposure variables by categorizing each exposure into low (reference), medium and high number of residents (supplementary table 2). For the between-periods within-worker effect, we found that periods where workers reached high numbers of residents per day (≥ 1 than the average for the worker across 14 months) were associated with a 22% increased risk of reporting ≥1 day with low-back pain per month (adjusted model: IRR 1.22, 95% CI 1.10–1.36), and an 8% increase in the mean low-back pain intensity (adjusted model: IRR 1.08, 95% CI 1.01–1.15) compared to periods with a low number of residents. Periods with a medium number of residents per day (-1–1 compared to the average across 14 months) were associated with a small and non-significant increase in the reported number of days with low-back pain (adjusted model: IRR 1.03, 95% CI 0.95–1.13) and intensity of low-back pain (adjusted model: IRR 1.02, 95% CI 0.96–1.08). This illustrates a non-linear trend, ie, a marked increased risk for low-back pain in periods when caring for a high number of residents compared to a low number of residents. However, there was no marked increase in low-back pain for periods with medium levels of exposure.

For the between-worker effect, caring for a high number of residents was associated with a 23% increased risk of reporting ≥1 day of low-back pain per month, and a 1% increase in the mean low-back pain intensity, but these associations were non-significant.

### Sensitivity analyses 2 - Number of residents per day (weighted) and low-back pain

In our second sensitivity analysis, we created weighted exposures accounting for the RNPA scale. Our results were similar to the main analyses, but with slightly weaker associations with low-back pain (supplementary table S3). The between-period, within-worker effect was associated with a 3% increased risk of reporting ≥1 day with low-back pain per month (adjusted model: IRR 1.03, 95% CI 1.01–1.04) and a 1% increase in the mean low-back pain intensity (adjusted model: IRR 1.01, 95% CI 1.00–1.02). However, the result for low-back pain intensity was not significant (P=0.09). For the between-worker effect, the results pointed in the same direction as for the main analyses but were not significant.

## Discussion

The main finding of our study is that caring for a higher number of residents in periods over 14 months is associated with an increased number of days and intensity of low-back pain. The multilevel analysis indicated that the between-period, within-worker association was statistically significant, independently of the average number of residents cared for between workers (within-ward) and between wards, when adjusting for individual level covariates. As such, these results support the 'even between-period, within-worker' organizational strategy to be a promising preventive approach. This suggests that more evenly distributing the number of residents to each worker across periods over 14 months might prevent overload and thereby low-back pain. As such, this suggests that a more even distribution of residents over time may reduce the risk of low-back pain as it reduces peaks of high exposure, which may cause overload and increase the risk of low-back pain. When organizing eldercare work, nursing homes could consider this preventive organizational strategy, but it should be tested through workplace interventions before being implemented in practice.

The strength of these within-worker associations was more pronounced for the number of days with low-back pain (4% increased risk) compared to the intensity of low-back pain (2% increased risk). This suggests that during periods when workers care for many residents per day, they face a higher risk of more days with low-back pain, while the risk for increased intensity of low-back pain only increases slightly.

Previous research has shown a positive association between staffing levels and worker injuries (32), while only one previous study showed a positive, but non-significant, association between staffing ratios and musculoskeletal pain (7). However, we are not familiar with previous studies investigating the association between staffing ratios and low-back pain in a study design separating between workers and between-period, within-worker effects using day-to-day measures of the number of residents cared for.

Our main finding – that periods of working with a larger number of residents than usual increases low-back pain – may be explained by a higher number of care handlings in these periods including both the physical (eg, awkward postures during handlings) (5) and psychosocial demands (eg, emotional demands), subsequently leading to an increased risk of low-back pain (6, 33, 34).

Regarding the magnitude of the between-period, within-worker associations, the effect sizes were relatively small, showing a 2% and 4% increased risk of for intensity and days of low-back pain, respectively, for each unit increase in the number of residents per day, which may be of limited clinical relevance.

However, larger effects sizes were found when categorizing the exposure into low, medium, and high numbers of residents (see supplementary table S2). As such, these results suggested a notable increased risk of low-back pain in periods over 14 months with high numbers of residents cared for per day (ie, caring for ≥1 resident per day above the mean value over 14 months). Specifically, workers had 22% increased risk of reporting ≥1 day with low-back pain and 8% increased risk for a higher low-back pain intensity per month in periods when caring for high numbers of residents (≥1 residents compared to the mean value over 14 months) compared with periods with low numbers of residents (≤-1 residents compared to the mean value over 14 months). Based on previous studies on low-back pain and outcomes such as sickness absence risk, these associations may be of clinical relevance (10, 35). These additional findings therefore suggest that the number of residents cared for does not need to be completely even across 14 months as long as it is kept relatively close to the mean over 14 months.

For the 'even between-worker, within-ward strategy', we found that workers caring for more residents than the ward average over 14 months did not have a statistically significantly increased risk of low-back pain. Even though our results persisted when adjusting for factors such as work ability, low-back pain and weekly work hours, it is likely that individual differences in the way workers respond to the workload may contribute to these non-significant findings. As such, uneven numbers of residents cared for between workers within a ward might not increase the risk for low-back pain if being tailored to the work ability of the eldercare workers.

We would highlight that this reasoning is speculative and needs to be specifically investigated in future studies. The less significant findings may also be attributed to fewer observations, and therefore lower statistical power, when comparing workers within wards (one value for each worker) instead of repeated measurements comparing periods within workers (up to five values for each worker).

Furthermore, we examined if the association between the number of residents cared for and low-back pain was influenced by the RNPA level. As residents’ requiring higher physical assistance may impose higher physical demands on workers, we expected a more pronounced risk of low-back pain when caring for more residents with a higher assistance need. However, when adjusting the analyses for the level of RNPA, the results remained similar to the findings in the main analyses (while being slightly weaker and the between-period, within-worker effect no longer being statistically significant for low-back pain intensity). These findings may be explained by a reduced sample size for the weighted score due to fewer observations for the RNPA scale, possibly leading to a reduced effect size. Another possible reason is that workers primarily caring for residents with higher needs for physical assistance may more consistently use assistive devices, which could reduce the physical workload for those workers.

Strengths and limitations

Our study has several strengths. Firstly, measuring the daily number of residents cared for using repeated day-to-day prospective work schedules and low-back pain measures over 14 months reduces common methods and recall biases for exposure and outcome variables and increases statistical power. Secondly, using repeated data from the RNPA scale, allows for a more nuanced examination of the long-term impact on pain of the number of residents cared for and the different RNPA levels, which are important for developing policies and practical recommendations. Thirdly, the potential issue of reversed causality – where existing low-back pain might influence the number of residents a person can care for rather than the other way around – was mitigated by adjusting for baseline pain and other potential confounders, as well as by the multiple follow-up measurements of both exposure and outcome.

Finally, the multilevel structure containing data from independent sources from several organizational levels is a major strength. Combined with the use of repeated measures, this study design allowed us to distinguish the effects of changes in exposure for the between- and within-person relationships. This is crucial to minimize biased results and incorrect conclusions (36). Only a few studies have explored the effect of changes in workload between workers (within-ward) and periods (within-worker) over time and their relation with pain (15, 17).

Some limitations should be addressed. First, as pain was self-reported, it may be prone to bias. In addition, focusing only on low-back pain could hide potential associations with pain from other body regions. However, we chose to focus on low-back pain as it is the most prevalent musculoskeletal health issue among Danish eldercare workers (9). Secondly, the work schedules did not cover the complete period of three months for each period, which was decided for the data collection to reduce participant burden. It is possible that the number of residents per day changed during each period, possibly leading to exposure misclassification as it may have resulted in an underestimation of the true association with low-back pain. Thirdly, the low proportion of male workers is representative of the eldercare sector but precluded us from doing stratified analyses by sex, which may be another limitation of the study. Moreover, although our findings may be applicable to other geographical areas internationally, Danish nursing homes are organized and governed in a way that might differ from nursing homes in other countries or regions and might, therefore, not be directly generalizable. Thus, we encourage similar studies to be conducted in other countries. Additionally, we find it important to mention that selection bias may have occurred as 45% of the original sample with information on the work schedules was excluded due to missing baseline characteristics or pain measures. This might lead to a healthy worker effect, potentially underestimating the association between staffing ratio and risk of low-back pain. However, the included sample aligns with those in previous DOSES studies, ie, participants completing the baseline questionnaire. Finally, there is a potential risk of residual confounding from unmeasured factors, such as "working day and night". However, we did not have complete data on day or night shift work during the study period, which would be required to adjust for this factor in the analyses of both between workers and between-period, within-worker effects. Aside from this, we included the variables that we considered most relevant based on theory and previous research of their association with both the exposure and the outcome of our study. We also considered the risk of overfitting important and, therefore, limited the number of variables included in the model.

### Practical implications

Our findings supported the organizational strategy of maintaining a consistent number of residents and workload for workers over a 14-month period to prevent low-back pain (the even between-period, within-worker strategy).

This strategy can potentially be implemented in the daily planning of eldercare work by using work schedules to evaluate the number of residents cared for and assigning a more stable number of residents to each individual worker over time. In addition to preventing low-back pain, implementing this strategy could potentially improve the quality of resident care through more consistency in the care situations so that workers to a greater extent provide care to the same residents (37, 38). However, this needs to be verified in future intervention studies.

### Concluding remarks

Our study found that eldercare workers in Danish nursing home wards are more likely to experience low-back pain during periods when they care for a higher number of residents over a 14-month period. These findings suggest that an organizational strategy of more evenly distributing the number of residents each worker cares for across this period could help prevent low-back pain. Additionally, this approach may enhance the quality of care by providing greater consistency in the residents assigned to each worker.

## Supplementary material

Supplementary material
