# Supplementary material for: Organizational strategies of eldercare work and health – Is the daily number of residents cared for over 14 months associated with back pain?
Source: Scand J Work Environ Health. 2025 Apr 27;51(3):191–200. doi: 10.5271/sjweh.4207 (PMC12067635; doi:10.5271/sjweh.4207)
Supplement: Supplementary material [file SJWEH-51-191-S001.pdf]

Organizational strategies of eldercare work and health – Is the daily number of residents cared for over 14 months associated with back pain?[1]

by Christian Tolstrup Wester, MSc,[2] Stavros Kyriakidis, MSc, Anders Dreyer Frost, MSc, Charlotte Diana Nørregaard Rasmussen, PhD, Andreas Holtermann, PhD, David M Hallman, PhD

1. Supplementary material
2. Correspondence to: Christian Tolstrup Wester, Department of Ergonomic and Physical Working Environment, The National Research Centre for the Working Environment, Lersø Parkallé 105, 2100 København Ø, Copenhagen, Denmark [E-mail: ctw@nfa.dk]

Supplementary table S1. Numbers of residents cared for per day, means across periods.

	Overall (N = 513[a])	
Residents cared for per day	Mean (SD)	Median [Min, max]
Worker level	4.3 (2.2)	3.7 [1.0, 15]
Ward level	4.4 (1.2)	4.3 [1.0, 12.5]
Worker-ward	-0.2 (1.9)	-0.5 [-4.6, 9.4]
Worker-ward mean (between worker effect)	0.2 (1.6)	-0.5 [-3.7, 5.5]
Worker-ward mean difference (between periods, within worker effect)	0.01 (1.0)	0.02 [-5.1, 6.0]

Worker level = Number of residents per day for workers, Ward level = Number of residents per day for workers in wards
Worker-ward = Difference between worker and ward level, Worker-ward mean = Worker-ward averaged across all periods
Worker-ward mean difference = Difference between worker-ward (changing between periods for each worker) and worker-ward mean (constant for each worker)

Supplementary table S2. Categorization of exposures ("low" = ≤ -1 (ref), "medium" = -1 to 1, and "high" = ≥ 1)

	Low back pain (days)		Low back pain (intensity)	
**Residents per day**	Crude model[a]	Adjusted model[b]	Crude model[a]	Adjusted model[b]
	IRR (95% CI)	IRR (95% CI)	IRR (95% CI)	IRR (95% CI)
Between-workers medium	0.94 (0.69 – 1.30)	1.04 (0.81 – 1.33)	0.89 (0.70 – 1.14)	0.95 (0.79 – 1.13)
Between-workers high	1.06 (0.71 – 1.58)	1.23 (0.87 – 1.75)	1.00 (0.95 – 1.29)	1.01 (0.78 – 1.31)
Between-periods medium	1.04 (0.95 – 1.14)	1.03 (0.95 – 1.13)	1.02 (0.96 – 1.08)	1.02 (0.96 – 1.08)
Between-periods high	1.23 (1.14 – 1.37)**	1.22 (1.10 – 1.36)**	1.08 (1.01 – 1.16)*	1.08 (1.01 – 1.15)*

IRR = Incidence Rate Ratio, CI = Confidence Interval (Significance levels: *** < 0.001, ** < 0.01, * < 0.05, ¤ < 0.1)
[a]Between-workers effect, between-periods within-worker effect, ward mean.
[b]Crude model + age, sex, BMI, smoking, work ability, weekly work hours, baseline low-back pain (number of days or intensity).
Mean values for each category (SD):
Between-workers: Low: -1.6 (0.5), medium: -0.3 (0.5), high: 2.7 (1.2)
Between-periods: Low: -1.7 (0.7), medium: 0.01 (0.5), high: 1.7 (0.7)

Supplementary table S3. Weighted score, accounting for the Resident's-Need-For-Physical-Assistance score. Association between residents per day weighted using the RNPA score, between-workers and between-periods, and low back pain (days and intensity) over 14 months at worker (N=476) and ward (N=111) levels.

	Low back pain (days)		Low back pain (intensity)	
	Crude model[a]	Adjusted model[b]	Crude model[a]	Adjusted model[b]
Residents per day	IRR (95% CI)	IRR (95% CI)	IRR (95% CI)	IRR (95% CI)

Between-workers	1.01 (0.96 – 1.06)	1.02 (0.98 – 1.07)	1.00 (0.96 – 1.03)	1.01 (0.98 – 1.04)
Between-periods	1.03 (1.01 – 1.04)**	1.03 (1.01 – 1.04)**	1.01 (1.00 – 1.02)¤	1.01 (1.00 – 1.02)

IRR = Incidence Rate Ratio, CI = Confidence Interval. (Significance levels: *** < 0.001, ** < 0.01, * < 0.05, ¤ < 0.1)
[a]Between-workers effect, between-periods within-worker effect, ward mean.
[2]Crude model + age, sex, BMI, smoking, work ability, weekly work hours, baseline low-back pain (number of days or intensity).
Note: Estimates are shown with a weight for the need for physical assistance level among the residents cared for.

Supplementary description 1

Mathematical example of the organizational exposures (between workers and between-periods within worker effects)
For each worker in each period, we calculated the exposures of 'the number of residents cared for per day' based on the following five steps:

Step 1: Worker mean (number of residents per day, worker level)
For example, during period 1, a worker cared for 80 residents during 21 workdays, resulting in a worker mean of 3.8 residents per day. This reflects the number of residents the particular worker cared for daily in that period.
Calculation for this step (in each period): $\frac{Total\ residents}{Total\ workdays} = residents\ per\ day$

Step 2: Ward mean (Number of residents per day, ward level)
For example, in period 1, a ward consists of three workers. The workers care for 3.8, 6.0 and 3.0 residents per day, respectively. This results in a ward mean of 4.3 residents per day ('between wards').
This reflects the average number of residents cared for per day among the workers in the ward in that period.
Calculation for this step (in each period): $\frac{Residents\ per\ day\ for\ each\ worker\ in\ the\ ward}{No.\ of\ workers\ in\ the\ ward} = ward\ mean;$

Step 3: Worker-ward (worker mean and ward mean difference, for each period)
To distinguish the variation in the number of residents per day between the worker mean and the ward mean we calculated the difference between the worker mean and ward mean. For example, a worker cares for 3.8 residents per day and the ward mean is 4.3 residents per day. This results in -0.5 residents per day ("worker-ward"); meaning that the worker cared for 0.5 residents per day less than the ward mean in that specific period.
Calculation for this step (for each period): $\frac{Residents\ per\ day}{Ward\ mean} =$ "worker-ward"

Step 4: Worker-ward mean across periods (Between-workers, within-ward) – exposure 1
To know the average difference in exposure between-workers across periods ("worker-ward mean"), we calculated this for each worker as the average worker-ward over five periods. Eg, a worker had a worker-ward of:
-0.5 residents/day in period 1
0.8 residents/day in period 2
1.5 residents/day in period 3
-1 residents/day in period 4 and
1.1 residents/day in period 5.
This results in a worker-ward mean of 0.38 residents per day for one worker across all periods. This means that this worker cared for averagely 0.38 residents per day more than the ward mean, across all five periods.
Calculation for this step: $\frac{Total\ worker-ward\ across\ periods}{Total\ number\ of\ periods} =$ "worker-ward mean across periods"

Step 5: Worker-ward mean difference (Between-periods, within-worker) – exposure 2
To understand the exposure change between periods for each worker, we calculated this as the difference between the worker-ward (in each period) and 'the worker-ward mean across periods'. For example, if 'the worker-ward mean across periods' is 0.38, and the worker-ward for each period is: -0.5 in period 1, 0.8 in period 2, 1.5 in period 3, -1 in period 4 and 1.1 in period 5, then the worker-ward mean differences for each period results in:
-0.12 residents/day in period 1,
0.42 residents/day in period 2,

1.12 residents/day in period 3,
-1.38 residents/day in period 4 and
0.72 residents/day in period 5.
This means that this worker in three periods cares for higher numbers of residents than the usual and in two periods the worker cares for lower numbers of residents than the usual number.
Calculation for this step (for each period): "worker-ward (minus) worker-ward mean" = "worker-ward mean difference"

Supplementary description 2
Mathematical example of the weighted number of residents cared for per day
Duration of care situations observed in the validation study were averagely 27.1 minutes for level 1 residents, 36.9 minutes for level 2, 51.7 minutes for level 3 and 67.8 minutes for level 4 as described by Jacobsen et al. (25). Thus, we calculated the weights for each level as the ratio duration of care between each of the upper levels and RNPA level 1 as the reference (weight = 1).

For example, the weight for level 2 was calculated as:

$$\frac{36.9\ minutes\ (level\ 2)}{27.1\ minutes\ (level\ 1)} = 1.36\ times\ the\ care\ duration\ of\ RNPA\ level\ 1$$

The weight of level 3 was: 1.91 and level 4: 2.5, respectively. Next, we computed the weighted score by multiplying the number of residents cared for with the corresponding number from the weights from each RNPA level (residents per day * weight). For instance, if a worker during a period cared for a total of 5 residents, where 3 residents had RNPA level 1 and 2 residents had RNPA level 3, the weighted score would be:

$$(3\ residents \times 1) \times (2\ residents\ \times 1.91) = 5.82\ residents\ per\ day$$
